# Acidic Peptizing Agent Effect on Anatase-Rutile Ratio and Photocatalytic Performance of TiO2 Nanoparticles

**DOI:** 10.1186/s11671-018-2465-x

**Published:** 2018-02-09

**Authors:** Hatem A. Mahmoud, Katabathini Narasimharao, Tarek T. Ali, Kamal M. S. Khalil

**Affiliations:** 1 Chemistry Department, Faculty of Science, Sohag University, P.O. Box 82524, Sohag, Egypt; 2 Chemistry Department, College of Science, University of Hail, Ha’il, 81451 Kingdom of Saudi Arabia; 3 Chemistry Department, Faculty of Science, King Abdulaziz University, P. O. Box, 80203, Jeddah, 21589 Kingdom of Saudi Arabia

**Keywords:** Photocatalysis, Degradation, Raman, NanoTiO_2_, Peptizing acid, Organic dyes

## Abstract

TiO_2_ nanoparticles were synthesized from titanium isopropoxide by a simple peptization method using sulfuric, nitric, and acetic acids. The effect of peptizing acid on physicochemical and photocatalytic properties of TiO_2_ powders was studied. The structural properties of synthesized TiO_2_ powders were analyzed by using XRD, TEM, N_2_-physisorption, Raman, DR UV-*vis*, FTIR, and X-ray photoelectron spectroscopy techniques. The characterization results showed that acetic acid peptization facilitated the formation of pure anatase phase after thermal treatment at 500 °C; in contrast, nitric acid peptization led to a major rutile phase formation (67%). Interestingly, the sample peptized using sulfuric acid yielded 95% anatase and 5% rutile phases. The photocatalytic activity of synthesized TiO_2_ nanoparticles was evaluated for degradation of selected organic dyes (crystal violet, methylene blue, and *p*-nitrophenol) in aqueous solution. The results confirmed that the TiO_2_ sample peptized using nitric acid (with rutile and anatase phases in 3:1 ratio) offered the highest activity for degradation of organic dyes, although, TiO_2_ samples peptized using sulfuric acid and acetic acid possessed smaller particle size, higher band gap energy, and high surface area. Interestingly, TiO_2_ sample peptized with nitric acid possessed relatively high theoretical photocurrent density (0.545 mAcm^−2^) and pore diameter (150 Å), which are responsible for high electron-hole separation efficiency and diffusion and mass transportation of organic reactants during the photochemical degradation process. The superior activity of TiO_2_ sample peptized with nitric acid is due to the effective transfer of photogenerated electrons between rutile and anatase phases.

**Electronic supplementary material:**

The online version of this article (10.1186/s11671-018-2465-x) contains supplementary material, which is available to authorized users.

## Background

Titanium dioxide (TiO_2_) is a widely known semiconductor material for its use in many applications, including solar energy conversion, pollution control, and photocatalysis [1–3]. TiO_2_ generally has three polymorphs, namely anatase, rutile, and brookite. It was reported that anatase and brookite can be transformed into rutile after thermal treatment at high temperature (< 610 °C) [4, 5]. TiO_2_ anatase is known to be an active photocatalyst for degradation of organic pollutants [1, 5–8]. It was observed that the size, crystalline phase, and porosity of the TiO_2_ samples have a strong influence over their applications [9]. Low-temperature synthesis of porous nanosized TiO_2_ requires longer synthesis times [10–12]. Li et al. [13] synthesized pure anatase and mixture of rutile and anatase phases by thermally treating the amorphous TiO_2_. The formation of pure anatase involves thermal treatment at high temperature (500 °C) [14], which often causes sintering of the TiO_2_ nanostructures. Synthesis of pure crystalline anatase at lower temperatures is an interesting topic of research [15]. Sol-gel and hydrothermal synthesis methods [16] were used to prepare a well-crystalline TiO_2_ at low temperature and short reaction time [17]. Wang et al. [12] synthesized highly crystalline anatase and rutile nanoparticles by hydrothermal HNO_3_ peptized TiO_2_ sols. However, the hydrothermal method demands special synthesis conditions and costly equipment which could bare high pH and temperatures [18].

The sol-gel synthesis method was used by utilizing the titanium alkoxide as Ti precursor at a mild temperature (< 100 °C), and it yielded highly dispersed nanosized TiO_2_ samples [16]. The chemical peptization method was adapted for the synthesis of stable metal oxide nanostructures including TiO_2_ [19], where the coagulated suspension dissolves and recrystallizes into the stable solution of nanoparticles with the peptizing agent [20]. It was reported that the nature of peptizing acid has an effect on the physicochemical characteristics such as crystallite size, composition, and morphology of particles [21]. Zaban et al. [22] synthesized TiO_2_ colloids with HNO_3_ and CH_3_COOH under hydrothermal conditions and observed the formation of anatase and brookite mixture in both cases. Liu et al. [23] obtained TiO_2_ hydrosol from metatitanic acid under different peptizing agents and studied the influence of peptizing conditions on the structural and photocatalytic properties of TiO_2_ hydrosols. Kanna and Wongnawa [24] employed sol-gel synthesis method to obtain amorphous-anatase-rutile by using different acids such as HCl, HNO_3_, H_2_SO_4_, H_3_PO_4_, and CH_3_COOH. The authors observed that presence of sulfate and phosphate groups are responsible for the inhibition of the growth of rutile phase. Later, Alphonse et al. [25] synthesized TiO_2_ aggregates, which are composed of anatase and brookite phase by hydrolysis of titanium isopropoxide in a highly acidic medium. Parra et al. [26] studied the reaction pathway in the synthesis of anatase nanoparticles with acetic acid. They used FTIR and NMR techniques to conclude that acetate ions act as bidentate ligand between two Ti centers.

Zhou et al. [27] studied the effect of HCl, HNO_3_, and CH_3_COOH in solvothermal method to synthesize 3D TiO_2_ structures with different morphology. The authors concluded that the sample synthesized with 0.68 M HCl possessed both anatase/rutile phases and offered highest photocatalytic activity due to its unique morphology and optical properties. Tobaldi et al. [28] adopted controlled hydrolysis/peptization of titanium isopropoxide with HNO_3_, HBr, and HCl to synthesize TiO_2_ nanoparticles. It was observed that halide ions enhanced the anatase-to-rutile phase transition, and the samples contained up to 77 wt% rutile and 5 wt% brookite after calcination at 450 °C.

In earlier publication [29], synthesis of nanosize TiO_2_ powders by acidic peptization of xerogels under atmospheric humidity conditions was performed. It was observed that the acidic peptization accompanied with ultrasonic vibrations has an effect on TiO_2_ structural properties. However, only few studies were devoted to study the effect of peptizing conditions on the formation of rutile phase and its subsequent effect on the photocatalytic activity of TiO_2_ nanoparticles. In this paper, we examined the influence of nature of peptizing acid (H_2_SO_4_, HNO_3_, and CH_3_COOH) on the formation of rutile phase and its influence in the photocatalytic efficiency of TiO_2_ nanoparticles in the degradation of three different organic pollutants (crystal violet (CV), methylene blue (MB), and *p*-nitrophenol (*p*-NP)).

## Methods

### Preparation of TiO2 Nanoparticles Using Different Peptizing Acids

Titanium tetra-isopropoxide [Ti(OPri)_4_] was used as Ti precursor, and the hydrolysis of Ti(OPri)_4_ was carried out under standard atmospheric conditions [29]. The typical synthesis procedure can be described as follows: 50 mL of Ti(OPri)_4_ was placed in a dark glass bottle, and the bottle was left in the fume hood for 15 days. The temperature and humidity of the fume hood were measured as 25 ± 5 °C and 50 ± 10%, respectively. The hydrolysis of the Ti precursor was completed in 15 days, and the resulted solution was transformed into a gel, which was then dried to obtain xerogel. The peptizing acid (100 mL of 1 N CH_3_COOH or HNO_3_ or H_2_SO_4_) was pipetted into a glass beaker, and the known amount of amorphous xerogel powder (2.0 g) was slowly added to the peptizing acid under constant stirring. Then, the beaker was placed in an ultrasonic bath which was maintained at 40 °C, and the mixture was subjected to ultrasonic treatment for 10 min. The peptized TiO_2_ nanoparticles were collected after centrifugation. Then, the materials were washed with distilled water and calcined for 3 h at 500 °C. The synthesized samples were labeled as TiO_2_ acronym prefix after their peptizing acid as “ace,” “nit,” and “sul” corresponding to CH_3_COOH, HNO_3_, and H_2_SO_4_, respectively.

### Material Characterization

The powder X-ray diffraction profiles were collected using a Philips PW1700 diffractometer with Cu Kα radiation and graphite monochromator with automatic divergent slit. The XRD profiles were indexed with standard JCPDS data. Spurr and Myers [30] formula [Eq. (1)] was used to determine the weight fractions of anatase and rutile phases.

1 $$ {X}_{\mathrm{R}}=1/\left[1+k\ \left({I}_{\mathrm{A}}/{I}_{\mathrm{R}}\right)\right] $$

where *I*_A_ and *I*_R_ are the integrated intensities of (101) reflection of anatase and (110) reflection of rutile, respectively. The empirical constant *k* was taken as 0.80 in this work. The crystallite size of the synthesized samples was measured using the Scherrer formula [Eq. (2)] and anatase (101) and rutile (110) reflections.

2 $$ D= B\lambda /{\beta}_{1/2}\cos \theta $$

where *D* is the average crystallite size of the phase, *B* is the Scherrer constant (0.89), *λ* is the wavelength of the X-ray radiation (1.54056 Å), *β*_1/2_ is the full width at half maximum of the reflection, and *θ* is the diffraction angle.

TEM analysis of the samples was carried out using Philips CM200FEG microscope equipped with a field emission gun at 200 kV. The coefficient of spherical aberration Cs = 1.35 mm was applied. HRTEM images with a pixel size of 0.044 nm were taken with a CCD camera. The laser Raman spectral analysis of the samples was carried out using Bruker Equinox 55 FT-IR spectrometer equipped with an FRA106/S FT-Raman module and a liquid N_2_-cooled Ge detector using the 1064-nm line of a Nd:YAG laser with an output laser power of 200 mW.

N_2_-physisorption measurements were carried using ASAP 2010 instrument, Micromeritics Instrument Corporation, USA. Specific surface area (*S*_BET_) of the samples were measured using N_2_-adsorption values and the BET equation. The pore width and the pore volume of the samples were determined by applying the BJH method.

Diffusive reflectance UV-*vis* spectra for synthesized TiO_2_ samples were recorded using Thermo Scientific Evolution spectrophotometer in the wavelength range of 220–700 nm. Band gap energy of the samples was determined using Kubelka-Munk transformation (*K*) as presented in Eq. (3).

3 $$ K=\frac{{\left(1-R\right)}^2}{2R} $$

where *R* is the reflectance. The wavelengths (nm) were translated into energies (eV), and a plot of ${\left(\mathrm{Kh}\upnu \right)}^{0.5}$vs. hν was drawn. The band gap energy (eV) was estimated as the intersection of the two slopes of the drawn curve.

The X-ray photoelectron spectra of the samples were collected using Thermo Scientific Escalab 250 Xi XPS instrument with Al Kα X-rays having a spot size of 650 mm. The peak shift due to charge compensation was corrected using the binding energy of C*1s* peak. The data was acquired using pass energy of 100 eV, dwell time 200 ms with a step size of 0.1 eV and 10–30 scans.

### Photocatalytic Degradation of Crystal Violet, Methylene Blue, and *p*-Nitrophenol

The photocatalytic degradation of CV, MB, and *p*-NP experiments were conducted in a glass reactor using synthesized TiO_2_ samples as a photocatalyst under UV irradiation for different reaction times. Total six black UV lamps (F20 T8 BLB) with 18 W power and 60 × 2.5 cm dimensions were used. The total power of the UV irradiation at the surface of an aqueous organic dye solution was measured with a Newport 918DUVOD3 detector, and power meter was measured as 13 Wm^−2^. One hundred milligrams of catalyst was added to 100 mL of aqueous organic pollutant (10 ppm) solution. Prior to the evaluation of photocatalytic efficiency of the catalyst, the organic dye solution was equilibrated with catalyst by stirring for 45 min to stabilize the adsorption of organic dye on the surface of the catalyst. The photocatalytic degradation of CV, MB, and *p*-NP was monitored by measuring the absorbance of organic dye at a regular time interval by using a Thermo Fisher Scientific Evolution 160 UV-*vis* spectrophotometer. The degradation percentage was calculated using the expression

4 $$ \eta =\left(1-C/{C}_0\right)\times 100 $$

Where *C*_0_ is the concentration of organic dye before illumination and *C* is the concentration after a certain reaction time.

The stability of the photocatalysts was analyzed by the reusability experiments. The regeneration of the catalyst was carried out using a simple procedure. After the first cycle of activity measurement, the catalyst was filtered from the photoreactor and the aliquots by centrifugation. The obtained catalyst was thoroughly washed with a distilled water and acetone. The catalyst was dried at 50 °C for 2 h and then reused for the next cycle of the photocatalysis measurements. Similarly, the experiment was repeated for several cycles to study the stability of the catalyst.

## Results and Discussion

### X-ray Powder Diffraction

**Fig. 1:**
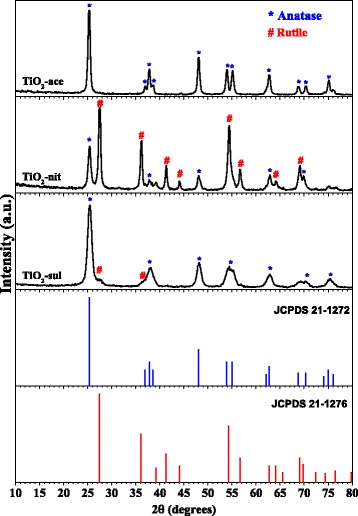
Powder XRD patterns of the calcined TiO_2_ samples (reprinted with permission from [29]. Copyright @ 2017 Elsevier)

The X-ray diffraction patterns of calcined TiO_2_-ace, TiO_2_-nit, and TiO_2_-sul samples are displayed in Fig. 1. The XRD peak positions and intensities for TiO_2_ phases presented in the samples are complimented with the JCPDS database. It is known that the anatase phase shows major diffraction peaks at 2*θ* values of 24.8°, 37.3°, 47.6°, 53.5°, 55.1°, and 62.2° matched to (101), (004), (200), (105), (211), and (204) crystal planes [JCPDS No. 21-1272]. On the other hand, the rutile phase shows major diffraction peaks at 2*θ* values of 27.0°, 35.6°, 40.8°, 54.0°, 53.9°, 56.1°, and 61.0° which corresponds to crystal planes of (110), (101), (200), (111), (210), (211), (220), (002), and (310) [JCPDS No. 21-1276]. The crystallite size and weight fractions of anatase and rutile phases presented in the samples were determined using Scherrer formula and Spurr and Myers method, respectively. The powder XRD pattern of TiO_2_-ace sample showed that it is composed of pure anatase phase (100%) with a particle size of 48 nm (Table 1).

**Table 1:** Results From XRD, TEM, and N_2_-physisorption Measurements

Sample	Anatase			Rutile			*S*_BET_(m^2^ g^−1^)	Pore Volume (cm^3^ g^− 1^)	Pore Diameter (Å)
	Phase (%)	Size (nm)		Phase (%)	Size (nm)				
		^a^TEM	^b^XRD		^a^TEM	^b^XRD			
TiO_2_-ace TiO_2_-nit TiO_2_-sul	100 33 95	20 17 7	48 41 23	0 67 5	– 15 8	– 51 –	115 36 50	0.243 0.069 0.192	83 150 72

^a^Particle size

^b^Crystallite size

The TiO_2_-sul sample possessed majorly anatase phase (95%) with particle size around 23 nm; however, a small diffraction peak corresponding to (110) plane of the rutile phase can be seen in this sample. In contrast, TiO_2_-nit sample showed XRD reflections for both anatase and rutile phases with a crystallite size of 41 nm and 50 nm, respectively. It is observed that the rutile is the major phase (67%) in this sample. These results indicating that nature of peptizing acid play a role in the formation of TiO_2_ phase.

### High-Resolution Transmission Electron Microscopy

**Fig. 2:**
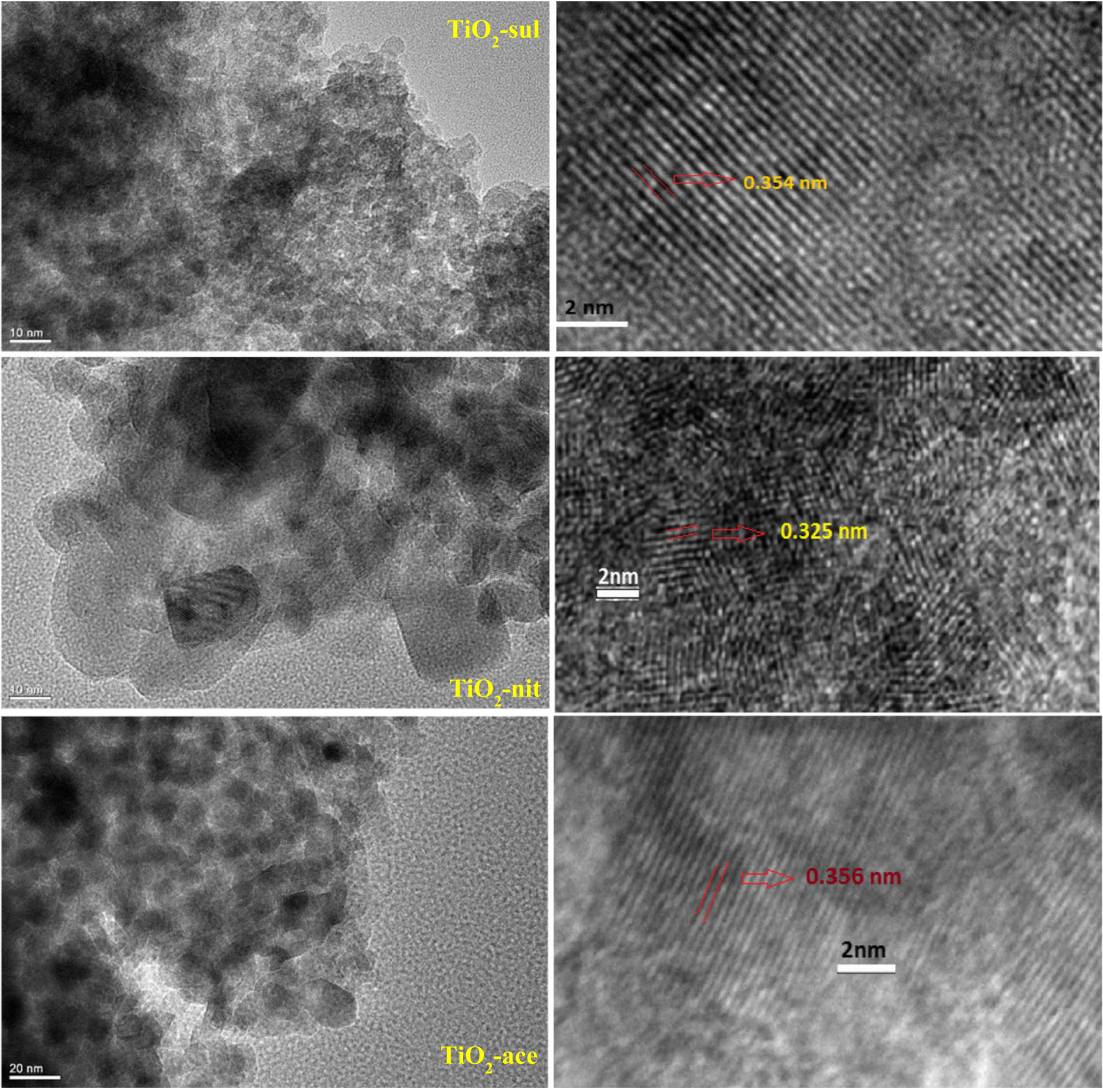
TEM and HRTEM images of the calcined TiO_2_ samples

TEM was performed to examine the size of the particles, crystallinity, and morphology of synthesized TiO_2_ nanopowders. The TEM and HRTEM pictures of synthesized TiO_2_ nanopowders are displayed in Fig. 2. It can be seen that the TiO_2_-sul sample consists closely packed agglomerated anatase particles with estimated average grain size about 7 nm. The TiO_2_-nit sample possessed nanoparticles sized between 10 and 20 nm with spherical morphology and also big sheets with 20 nm width. In contrast, TiO_2_-ace sample consists of TiO_2_ nanoparticles (15–20 nm) consisted of mostly defined spherical morphology. Vinogradov and Vinogradov [31] also observed the similar type of results that the small size aggregates were detected when strong peptizing acids such as HNO_3_ and H_2_SO_4_ were used for the peptization. The crystallite size measured by Scherer’s formula resulted in larger crystallite sizes compared to grain size measured with TEM analysis. As reported previously, crystallite size is different from grain size; however, crystallite size could match with the grain size in some cases [32]. It can be observed that the HRTEM images of TiO_2_-sul and TiO_2_-ace samples showed particles that contained fringes corresponding to anatase crystal lattice planes with *d*-spacing of 0.356 nm for the (101) plane [33], whereas the HRTEM image of TiO_2_-nit sample showed particles with lattice fringes for rutile crystal lattice plane (110) with *d*-spacing of 0.325 nm along with the anatase crystal lattice (101) plane.

### Raman Spectroscopy

**Fig. 3:**
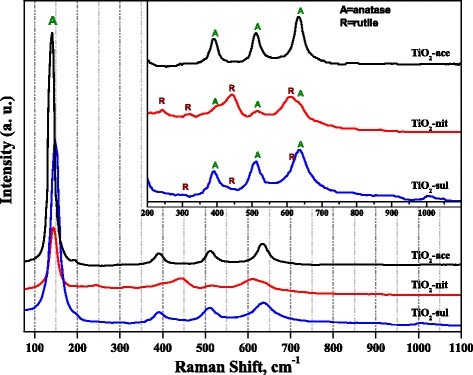
Raman spectra of the calcined TiO_2_ samples

Raman spectroscopy was also used to probe the phase formation in the synthesized TiO_2_ samples. Figure 3 shows Raman spectra obtained for the three TiO_2_ samples calcined at 500 °C. It is reported that anatase and rutile phases possessed six and five active Raman bands, respectively, (anatase 143, 195, 395, 512, and 638 cm^−1^; rutile 145, 445 , 611, and 826) [34]. It is clear from Fig. 3 that all three samples showed highly intense sharp Raman band (*E*_g_) in the range of 141–146 cm^−1^, which is characteristic band due to the presence of anatase phase. Low intense Raman bands due to both anatase and rutile phases can be observed clearly in the inset figure. The TiO_2_-nit and TiO_2_-sul samples showed Raman bands due to both anatase and rutile phases; however, the intensity of Raman bands due to the presence of rutile phase is high in case of TiO_2_-nit sample. In contrast, TiO_2_-ace sample exhibited Raman bands due to anatase phase only.

It was reported that Raman spectroscopy results could be used to investigate the particle size of TiO_2_ nanoparticles, since an unusual band shifts of Raman bands could be correlated to decrease of particle size of the samples [35]. In Fig. 3, the TiO_2_-ace sample exhibited *E*_g_ band at 141.5 cm^−1^; however, the band was shifted to 146 and 150 cm^−1^ in case of TiO_2_-nit and TiO_2_-sul samples, respectively. The observations from Raman spectra indicate that TiO_2_-sul sample possessed small particle size than the other two samples, which is in accordance with XRD and TEM observations.

### Diffuse-Reflectance UV-*vis*

**Fig. 4:**
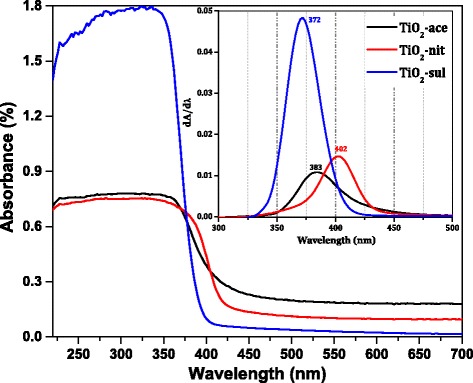
DR UV-*vis* spectra of the calcined TiO_2_ samples (inset; the derivative of the DR UV-*vis* spectra)

The DR UV-*vis* spectra of the synthesized TiO_2_ samples thermally treated at 500 °C were shown in Fig. 4. The position of the peak maximum in the derivative of the DR UV-*vis* spectra for three samples was shown in the inset of the figure. It clearly shows that the samples have a strong electronic reflectance in the UV region. The reflectance peak maximum is different for the samples synthesized using three different acids. The TiO_2_-sul sample showed peak maximum at 372 nm, while it is shifted to 383 nm in TiO_2_-ace and 402 nm for TiO_2_-nit sample, respectively. It is reported that the anatase and rutile have band gap energy of 3.2 eV (380 nm) and 3.0 eV (415 nm), respectively [1]. The differences in reflectance maximum could be attributed to the change of crystallite size and phase structure of the samples [36]. The absorption maximum shifted toward higher wavelengths for the samples which have more percentage of rutile phase. The band gap energy (eV) was calculated for the calcined samples by determining the relation between hν and (αhν) [2] [Additional file 1: Figure S1]. The data revealed that the band gap energy for TiO_2_-sul (3.12 eV) is higher compared to TiO_2_-ace (2.99 eV) and TiO_2_-nit (2.97 eV). The band gap of TiO_2_ decreased when the rutile phase is dominated in the sample. It was reported that the valence band (VB) of anatase and rutile phases is majorly due to O*2p* states; on the other hand, the conduction band (CB) is composed of Ti *3d* states [37]. The band gap energy of TiO_2_ is established by the CB and VB positions, which is majorly influenced by phase composition. So, the band gap energy of the sample which contained both anatase and rutile phases should be in between of the values of pure anatase and rutile.

### N_2_-Physisorption Measurements

**Fig. 5:**
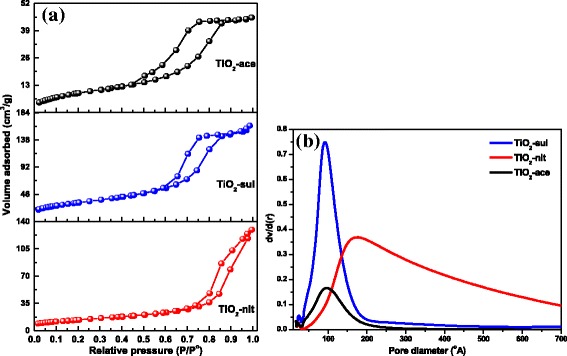
**a** N_2_ adsorption-desorption isotherms. **b** Pore size distribution for the calcined TiO_2_ samples (reprinted with permission from [29]. Copyright @ 2017 Elsevier)

Nitrogen adsorption-desorption isotherms for the three synthesized samples are presented in Fig. 5a. Type-IV isotherms with H2-type hysteresis loop was observed for the samples synthesized by peptization with acetic acid (TiO_2_-ace) and sulfuric acid (TiO_2_-sul). This indicates that these two samples possess mesopores resulted from the aggregates of TiO_2_ nanoparticles. However, typical type-IV isotherm with narrow H3-type hysteresis loop (characteristic of open and/or slit-shaped pores) was observed for TiO_2_-nit sample. It can also be observed that the hysteresis loop was closed at high relative pressure (*P/P*^0^ *=* 1) and this observation indicating the presence of pores with large size [38].

The BJH model pore size distributions for the synthesized materials were obtained from the values of adsorption branch of the isotherms. The BJH pore size distributions of the samples are shown in Fig. 5b. Narrow monomodal pore size distributions were observed for TiO_2_-ace and TiO_2_-sul samples. However, wider pore diameter distribution was observed for the sample prepared with nitric acid probably due to void spaces existed in between larger particles. Textural properties of the samples are presented in Table 1. Results showed that high surface area (115 m^2^ g^−1^) was observed for the TiO_2_-ace sample calcined at 500 °C. The order of *S*_BET_ change was TiO_2_-ace > TiO_2_-sul > TiO_2_-nit. The observed results clearly indicate that the adapted peptization conditions were very effective in the generation of nanoparticles with porous texture.

### Fourier Transform Infrared Spectroscopy

**Fig. 6:**
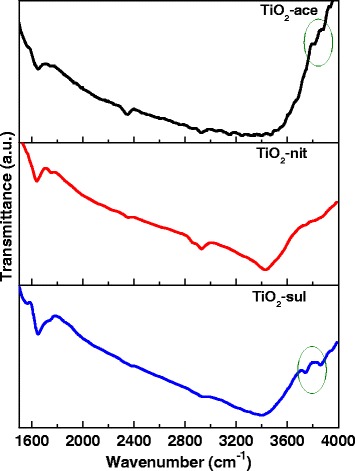
FTIR spectra of the calcined TiO_2_ samples

The photocatalytic activity of TiO_2_ depends upon crystallinity, crystallite size, composition, electron-hole recombination rate, surface area, and also the density of surface hydroxyl groups [39]. FTIR and XPS spectroscopic techniques were used to investigate the nature of –OH groups presented in the calcined TiO_2_ samples. Figure 6 shows FTIR spectra for the three TiO_2_ samples in the range of 1600–4000 cm^−1^. It is reported that TiO_2_ support could possess different types of surface hydroxyl groups; they can be categorized as isolated Ti-OH, hydroxyl groups bonded one to another via hydrogen bonding and chemically bonded H_2_O molecules [40].

The three samples showed a broad band centered at 3408 cm^−1^, which is attributed to the stretching vibration of O-H groups (water molecules and the free surface –OH groups). Additional bands also appeared at 2340 and 1640 cm^−1^, which could be assigned to O-H stretching vibration and molecularly adsorbed H_2_O, respectively [41]. An existence of two –OH stretching vibrations in case of anatase (at 3715 and 3675 cm^−1^) and one weak band at 3680 cm^−1^ with rutile were reported previously [42]. A very similar result can be seen in case of synthesized TiO_2_ samples.

### X-ray Photoelectron Spectroscopy

**Fig. 7:**
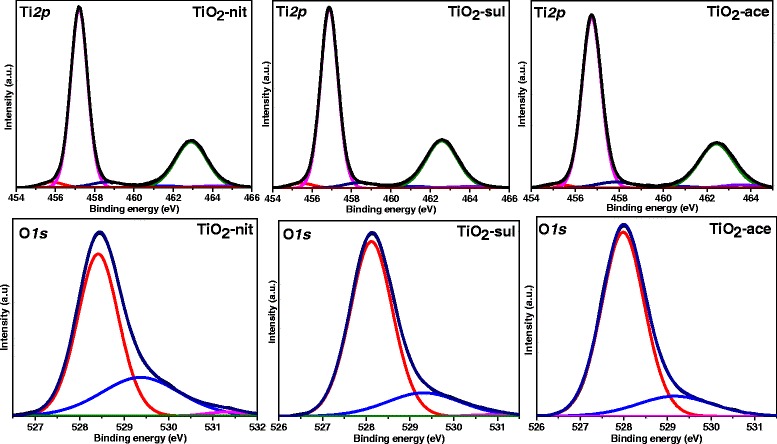
Ti *2p* and O*1s* X-ray photoelectron spectra for the calcined TiO_2_ samples

Figure 7 shows deconvoluted Ti*2p* and O*1s* XP spectra for synthesized TiO_2_ samples. The three samples showed two major peaks at 457.2 and 463.1 eV corresponding to *2p*_*3/2*_ and *2p*_*1/2*_ of Ti^4+^ in TiO_2_ [43]. Very similar binding energy values were observed in Ti *2p* region for all the three TiO_2_ samples indicating that the Ti atoms in these samples existed in the same oxidation state. Two small shoulder peaks at 455.8 and 458.7 eV were also observed for all the samples. The shoulder at 455.8 eV could be assigned to a Ti^3+^ state, due to an oxygen deficiency in TiO_2_ [44], while the other shoulder peak at 458.7 eV arises from a Ti^4+^ state of the Ti-OH species [45]. It is clear from the Ti*2p* spectra that the contribution of oxygen-deficient TiO_2_ species is higher in TiO_2_-nit than TiO_2_-sul and TiO_2_-ace samples. All the samples showed O*1s* XP peaks at 528.4, 529.3, and 531.3 eV. The XPS peak at 528.4 eV can be attributed to O-Ti^4+^ species in the TiO_2_ crystal lattice, while other two peaks at 529.3 and 531.3 eV can be assigned to oxygen species presented in surface adsorbed hydroxyl groups [46].

McCafferty [47] also observed that the O*1s* peak possessed a tail peak at the high binding energy values, which could be due to the presence of Ti-OH groups. Since the physically adsorbed surface, Ti-OH groups can be easily removed under the ultra-high vacuum used to operate the XPS instrument [48]. These –OH groups presented in the samples must be due to Ti-OH which are chemically bonded to the surface defects of TiO_2,_ where the percentages of –OH groups in total oxygen species for TiO_2_-nit sample are slightly higher than that of TiO_2_-sul and TiO_2_-ace (Table 2).

**Table 2:** Surface Elemental Composition of the Samples From XPS Analysis

Sample	Elemental Surface Composition (XPS)				
	Total Ti *2p* Species	O *1 s* (eV)			
		O-Ti^4+^ (528.4)	Ti-OH (529.3)	Ti-OH (531.3)	Total
TiO_2_-nit TiO_2_-sul TiO_2_-ace	56.3 57.8 57.2	33.0 31.8 34.0	9.5 9.4 8.4	1.2 1.0 0.4	43.7 41.7 42.8

### Photocatalytic Degradation of Crystal Violet, Methylene Blue, and *Para*-nitro Phenol Dyes

The photocatalytic activity of calcined TiO_2_ nanoparticles for the degradation of CV, MB, and *p*-NP was estimated. It is reported that photocatalytic degradation reaction generally follows Langmuir-Hinshelwood kinetics [1]. Hence, the photocatalytic degradation of organic dyes can be expressed as

5 $$ -\mathrm{dc}/\mathrm{dt}=\mathrm{kC} $$

and after the integration, Eq. (4) can be derived

6 $$ C={C}_0{\exp}^{\left(-\mathrm{kt}\right)} $$

where *C*_0_ is the initial concentration (ppm) of the organic dyes, and *k* is the rate constant, which depends on reaction time, temperature, and solution pH. Normally, the photocatalytic efficiency of the catalyst increases with time on stream.

Blank experiments were performed to confirm the significance of both photocatalyst and UV irradiation. No reaction was proceeded when the catalyst and UV irradiation applied alone. Similar results were observed in our previous findings [49]. As notified in the experimental section, the TiO_2_ photocatalysts were equilibrated with the organic dye solution for 45 min to determine the adsorption of organic dyes on synthesized TiO_2_ samples. The UV-*vis* absorption spectra of CV, MB, and *p*-NP were recorded after the equilibration of the photocatalyst. Additional file 1: Figure S2, S3, and S4 displayed the variation in the UV-*vis* absorbance spectra of CV, MB, and *p*-NP solutions (10 ppm) with different reaction times over TiO_2_-ace, TiO_2_-sul, and TiO_2_-nit samples, respectively. The intensity of absorption peaks which corresponds to the CV, MB, and *p*-NP was decreased with the increase of reaction time. The UV-*vis* spectra of reaction products indicate that organic dyes were degraded during the photoreaction. The TiO_2_-nit sample found to be the most effective photocatalyst in comparison with TiO_2_-sul and TiO_2_-ace samples. Degradation of 50% of *p*-NP was observed within 60 min for the TiO_2_-nit sample, whereas 75 and 100 min were needed for the degradation of 50% *p*-NP for the TiO_2_-ace and TiO_2_-sul samples under similar conditions. Similar photocatalytic activity patterns were observed for degradation of MB and CV dyes.

**Fig. 8:**
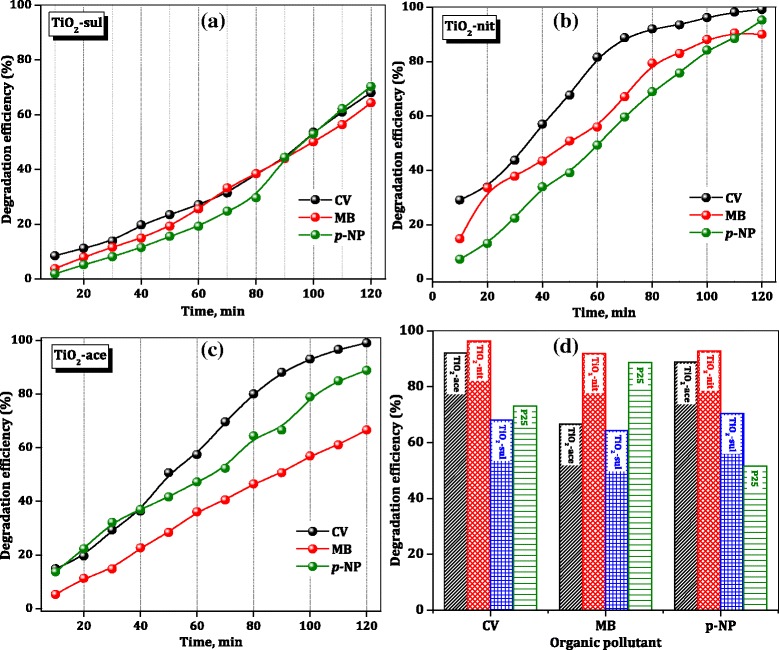
Photocatalytic degradation efficiency of TiO_2_ catalysts

The percentage degradation efficiency of the investigated catalysts was calculated using Eq. (4). Figure 8 shows the percentage changes of CV, MB, and *p*-NP aqueous solution at room temperature in the presence of calcined TiO_2_ samples. After just 10 min of the reaction, the TiO_2_-nit sample showed 29% CV degradation efficiency, while TiO_2_-ace and TiO_2_-sul samples showed only 17 and 9%, respectively. The photocatalytic activity steeply increased with the increase of reaction time over the three samples. However, after 120 min, TiO_2_-nit and TiO_2_-ace samples showed 99% efficiency; however, TiO_2_-sul sample showed only 65% efficiency.

To compare the photocatalytic performance of synthesized TiO_2_ samples, the degradation efficiency of the commercial P25 sample for organic dyes after 120 min is included in Fig. 8d. It is clear that TiO_2_-nit sample showed better performance than the P25 sample in the degradation of three organic dyes; however, TiO_2_-ace and TiO_2_-sul samples showed lower activity than the P25 catalyst in case of *p*-NP degradation. These results are suggesting that the performance of catalysts is influenced by the physicochemical characteristics of the TiO_2_ samples and nature of the organic dye.

The rate constants for photocatalytic degradation of CV, MB, and *p*-NP over synthesized TiO_2_ samples and commercial P25 sample were determined from the slope of the straight line which is plotted between ln(*C*_0_/*C*_t_) and *t*, and the results are presented in Table 3. The observed results are indicating that the photocatalytic activity of the degradation of organic dyes was greatly influenced by the composition of TiO_2_ sample and amount of the surface hydroxyl groups. The activity indeed is not influenced by the particle size, crystallinity, and surface area of TiO_2_ synthesized in this work. This observation is not consistent with the results observed by Fujishima et al. [8] that the catalyst which possesses lower particle size offered high photocatalytic efficiency.

**Table 3:** Rate Constants for Photocatalytic Degradation of Different Organic Compounds Over TiO_2_ Samples

Catalysts	Rate constants (min^−1^)		
	CV	MB	*p*-NP
TiO_2_-ace	0.0067	0.0023	0.0051
TiO_2_-nit	0.0316	0.0065	0.0157
TiO_2_-sul	0.0026	0.0035	0.0018
P25	0.0082	0.0051	0.0012

Previously, it was reported that anatase is a better photocatalyst than rutile due to its high band gap energy and a large number of surface OH groups [50]. It was thought that TiO_2_-nit sample would offer low photocatalytic activity due to the presence of more rutile phase (67%). However, Masahashi et al. [51] claimed that rutile exhibited higher performance than anatase MB degradation due to its superior crystalline nature.

Determination of photocurrent values was carried out to obtain a better insight responsible factor for the superior photocatalytic performance of the samples containing more rutile. It was reported that photocatalytic activity is directly related to the electron-hole separation efficiency of a catalyst which is influenced by the photocurrent density [52]. Theoretical photocurrent density of the TiO_2_ samples was calculated from the absorption edge of the TiO_2_ samples (obtained from DR UV-*vis* spectroscopy measurements) and theoretical equations (supporting information) presented in the literature [53]. The results of photocurrent of TiO_2_ samples are presented in Table 4 along with the percentage of rutile and photocatalytic efficiency values. The photocurrent density of TiO_2_-nit (0.545 mA/cm^2^) is higher than other two synthesized TiO_2_ samples and also commercial P25 sample (0.401 mA/cm^2^), manifesting the beneficial role of rutile phase in improving the photoactivity of TiO_2_ samples.

**Table 4:** Relation Between Rutile Percentage, Theoretical Photocurrent Density and Degradation Efficiency

Sample	^a^Rutile (%)	^b^Photocurrent Density (mA/cm^2^)	^c^Degradation efficiency (%)		
			CV	MB	*p*-NP
TiO_2_-ace	0	0.231	92.1	66.6	88.8
TiO_2_-sul	5	0.346	68.0	64.4	70.4
TiO_2_-P25	25	0.401	73.1	88.7	51.6
TiO_2_-nit	67	0.545	96.4	91.8	92.7

^a^Spurr and Myers method

^b^Theoretical photocurrent density calculations using DR UV-*vis* measurements

^c^Reaction time = 120 min, 25 °C, pollutant concentration = 10 ppm

Previously, Melcher et al. [54] reported that photocatalytic capability of the commercial P25 material originates due to the presence of a mixture of rutile and anatase phases in the sample (75% anatase and 25% rutile). Hirakawa et al. [55] indicated that pure rutile itself is not a powerful photocatalyst, and it is also reported that the light with a wavelength of 380 nm is not powerful enough to generate charge carriers in the pure anatase [56]. Based on XPS spectroscopy results and theoretical calculations, Scanlon et al. [57] concluded that electrons were moved from rutile to anatase and the holes were transported from anatase to rutile, which inhibited the electron-hole recombination. Yu et al. [58] reported a similar observation that TiO_2_ sample with mixed phases was beneficial to decrease the rate of h^+^-e^−^ recombination and thus enhance the photocatalytic efficiency of the catalyst.

**Fig. 9:**
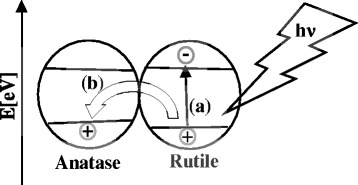
Plausible model of **a** generation of electron-hole pairs and **b** effective charge carrier separation via transfer of the generated holes into the anatase valence band

In literature reports, two possible transfer mechanisms have been proposed for anatase-rutile composite samples [59]. The first mechanism is the interfacial electron transfer from CB of anatase to that of the rutile [60], and the second one is an electron transfer from CB of rutile to lower energy anatase active sites [61]. It is known that the anatase CB possesses higher negative potential than the rutile CB due to the fact that anatase has a higher band gap (3.12 eV) than rutile. Therefore, it is not possible for an electron to move from the rutile CB to the anatase CB because it would have to overcome the energetic barrier between the two bands. The band gap of anatase VB is also slightly higher, than the rutile VB, so the generated holes could be moved to the anatase VB, to achieve an effective charge separation. Most probably, the electron-hole pair is formed in the composite of rutile and anatase in case of TiO_2_-nit and TiO_2_-sul samples (Fig. 9), and this rate is much higher in TiO_2_-nit sample due to predominant rutile formation.

It was reported that increase of crystal growth of initial phase is possible by increasing the mobility of ions presented in precursor solution [62]. Several researchers added small volumes of mineral acids (such as hydrochloric acid and sulfuric acid) to improve the mobility of dissociated ions [63]. Their role is not only to increase the rate of diffusion of ions in a solution but also to alter the surface charge. Under humidity conditions, titanium isopropoxide can subsequently undergo hydroxylation and polymerization to TiO_2_.

7 $$ \mathrm{Ti}{\left(\mathrm{OPri}\right)}_4+4{\mathrm{H}}_2\mathrm{O}\to \mathrm{Ti}{\left(\mathrm{OH}\right)}_4+4\ \mathrm{PriOH}\kern2.5em \left(\mathrm{hydroxylation}\right) $$

8 $$ \mathrm{Ti}{\left(\mathrm{OH}\right)}_{4\kern0.5em }\to {\mathrm{TiO}}_{2.}x{\mathrm{H}}_2\mathrm{O}+\left(2\hbox{-} x\right)\ {\mathrm{H}}_2\mathrm{O}\kern5em \left(\mathrm{condensation}\right) $$

Depending on the nature of peptizing acid, the transformation of TiO_2_ leads to anatase or rutile phase [64]. Formation of amorphous TiO_2_ or metastable anatase phase was observed when the condensation initiated before hydrolysis of Ti precursor. Under highly acidic conditions, the rutile phase formation is favorable as the rate of condensation is slow. Accordingly, the rutile phase was obtained when sulfuric and nitric acids were used for the peptization. The use of weak acid (acetic acid) as a peptizing agent allows the control of both the degree of condensation and oligomerization and persuades the preferential crystallization of TiO_2_ in the anatase phase. Zeng et al. [20] used polycarboxylic acid as a peptizing agent and observed the formation of nanoparticles of anatase which they attributed to chelation effect of organic acid.

It is known that TiO_6_ octahedra are a fundamental structural unit for both anatase and rutile phases (*D*_4h_ system), and the only difference between these two structures is the assembly of the octahedral chains [65]. Face-shared linking of TiO_6_ units results in anatase structure, while edge-shared linking results in rutile structure [66]. It is clear that NO^3−^ anions possessed weaker affinity to Ti atoms in an aqueous solution than CH_3_COO^−^ and SO_4_^2−^ anions. The strong affinity of CH_3_COO^−^ and SO_4_^2−^ anions with Ti atoms is responsible for the inhibition of the phase transformation.

In the previously reported studies, many of the photocatalysts have not been tested for reuse mainly due to undergo photocorrosion; hence, their photostability is reduced for further usage. The reusability of the calcined TiO_2_ samples was examined to study the effectiveness of these photocatalysts. It was observed that the used photocatalyst offered 90% efficiency for three consecutive cycles. The efficiency of the catalyst was reduced to 80 and 75% during fourth and fifth cycle, respectively. The decrease is due to the loss of some amount of catalyst during the filtration and regeneration procedures.

## Conclusions

A simple peptization method was adapted to synthesize TiO_2_ nanoparticles by using sulfuric, nitric, and acetic acid as peptizing agents and titanium isopropoxide as Ti precursor. The influence of acid species on the crystal phase, morphology, textural, and surface composition of TiO_2_ were studied in detail. The TiO_2_ sample peptized with acetic acid possessed pure anatase phase, while the formation of minor (5%) and major (67%) of rutile phase was observed in case of samples peptized with sulfuric acid and nitric acid, respectively. It is observed that TiO_2_ peptized with nitric acid showed sheet-like structures along with nanoparticles, while TiO_2_ samples peptized with sulfuric and acetic acids possessed near spherical nanoparticles. The photocatalytic properties of synthesized TiO_2_ nanostructures were evaluated for photodegradation of aqueous CV, MB, and *p*-NP solutions. The TiO_2_ peptized using nitric acid showed the best photocatalytic activity than commercial P25 and other two peptized samples, and its photodegradation efficiency was reached to 95% in 120 min for *p*-NP degradation. Although TiO_2_ samples peptized using sulfuric acid and acetic acid possessed smaller particle size, higher band gap energy, and high surface area, TiO_2_ sample peptized with nitric acid possessed a higher percentage of rutile and photocurrent density. The observed photocurrent density is dominated by the photoactivity of TiO_2_. The results indicate a direct correlation between the photocatalytic activity and the photocurrent density of the TiO_2_ samples. The superior activity of TiO_2_ sample peptized with nitric acid is due to the effective transfer of photogenerated electrons between rutile and anatase phases, and large pore diameter could have enhanced the diffusion and mass transportation of reacting molecules and OH radicals during the photochemical reaction. The synthesized TiO_2_ photocatalysts can be recycled with a minor change in the activity.

## Additional file

Additional file 1: Figure S1. The plots represent the relation between E and (K*E)^0.5^ for three samples. **Figure S2.** UV-*vis* absorption changes of aqueous solution of different organic compounds at 25 °C in the presence of TiO_2_-ace sample. **Figure S3.** UV-*vis* absorption changes of aqueous solution of different organic compounds at 25 °C in the presence of TiO_2_-nit sample. **Figure S4.** UV-*vis* absorption changes of aqueous solution of different organic compounds at 25 °C in the presence of TiO_2_-sul sample. Calculation of the theoretical photocurrent in TiO_2_ nanoparticles. (DOCX 768 kb)

## Abbreviations

CV: Crystal violet
DR UV-*vis*: Diffuse-reflectance ultraviolet-visible spectroscopy
FTIR: Fourier transform infrared spectroscopy
HRTEM: High-resolution transmission electron microscopy
MB: Methylene blue
NMR: Nuclear magnetic resonance
*p*–NP: *para*–nitro phenol
TEM: Transmission electron microscopy
TiO_2_: Titanium oxide
XPS: X-ray photoelectron spectroscopy
XRD: X-ray powder diffraction
