# Supplementary material for: Acidic Peptizing Agent Effect on Anatase-Rutile Ratio and Photocatalytic Performance of TiO2 Nanoparticles
Source: Nanoscale Res Lett. 2018 Feb 9;13:48. doi: 10.1186/s11671-018-2465-x (PMC5807258; doi:10.1186/s11671-018-2465-x)
Supplement: Supplementary file 1 — The plots represent the relation between E and (K*E)0.5 for three samples. Figure S2. UV-vis absorption changes of aqueous solution of different organic compounds at 25 °C in the presence of TiO2-ace sample. Figure S3. UV-vis absorption changes of aqueous solution of different organic compounds at 25 °C in the presence of TiO2-nit sample. Figure S4. UV-vis absorption changes of aqueous solution of different organic compounds at 25 °C in the presence of TiO2-sul sample. Calculation of the theoretical photocurrent in TiO2 nanoparticles. (DOCX 768 kb) [file 11671_2018_2465_MOESM1_ESM.docx]

**Additional file 1**

**Fig. S1**: The plots represents the relation between E and (K*E)^0.5^ for three samples

**Fig. S2:** UV-vis absorption changes of aqueous solution of different organic compounds at 25°C in the presence of TiO_2_-ace sample

**Fig. S3:** UV-vis absorption changes of aqueous solution of different organic compounds at 25°C in the presence of TiO_2_-nit sample

**Fig. S4:** UV-vis absorption changes of aqueous solution of different organic compounds at 25°C in the presence of TiO_2_-sul sample

**Calculation of the theoretical photocurrent in TiO_2_ nanoparticles**

The single photon energy is calculated from Equation S1

$E\left( \lambda\right)=h x \frac{C}{\lambda}$ (S1)

Where *E(λ)* is the photon energy (J), *h* is Planck’s constant (6.626×10^-34^ J s), *C* is the speed of light (3×10^8^ m s^-1^) and *λ* is the photon wavelength (m).

The UV photon flux is then calculated according to Equation S2

$Flux \left( \lambda\right)= \frac{P (\lambda)}{E (\lambda)}$ (S2)

Where *Flux(λ)* is the UV light photon flux (m^-2^ s^-1^ nm^-1^), and *P(λ)* is the UV light flux (W m^-2^ nm^-1^).

The theoretical maximum photocurrent density under UV light illumination, *J_max_* (A m^-2^), is then calculated by integrating the UV photon flux, shown in Equation S3:

$J_{max}=e x\int_{\lambda2}^{\lambda1} Flux \left( \right)d$ (S3)

Where *λ_1_* is the absorption edge of TiO_2_, *λ_2_* is the lower limit of the UV irradiation, and *e* is the elementary charge of electron (1.602×10^-19^ C).

The theoretical photocurrent for synthesized TiO_2_ samples were calculated accordingly.
